# [Retracted] Emodin alleviates gemcitabine resistance in pancreatic cancer by inhibiting MDR1/P‑glycoprotein and MRPs expression

**DOI:** 10.3892/ol.2024.14568

**Published:** 2024-07-15

**Authors:** Hongchun Guo, Feng Liu, Shuguang Yang, Tao Xue

Oncol Lett 20: 167, 2020; DOI: 10.3892/ol.2020.12030

Following the publication of this paper, it was drawn to the Editor's attention by a concerned reader that certain of the immunohistochemical staining data shown in Fig. 1E on p. 4 were strikingly similar to data that had already been submitted for publication in different form in another article written by different authors at different research institutes [Zheng T-L, Li D-P, He Z-F and Zhao S: miR-145 sensitizes esophageal squamous cell carcinoma to cisplatin through directly inhibiting PI3K/AKT signaling pathway. Cancer Cell Int 19: 250, 2019].

Owing to the fact that the contentious data in the above article had already been submission for publication prior to its submission to *Oncology Letters*, the Editor has decided that this paper should be retracted from the Journal. The authors were asked for an explanation to account for these concerns, but the Editorial Office did not receive a reply. The Editor apologizes to the readership for any inconvenience caused.

